# A review on nutritional quality of animal and plant-based milk alternatives: a focus on protein

**DOI:** 10.3389/fnut.2024.1378556

**Published:** 2024-07-05

**Authors:** Romdhane Karoui, Inès Bouaicha

**Affiliations:** Univ. Artois, Univ. Lille, Univ. Littoral Côte d’Opale, Univ. Picardie Jules Verne, Univ. de Liège, INRAE, Junia, Lens, France

**Keywords:** animal dairy products, plant-based milk alternatives, protein, nutritional, quality

## Abstract

In recent years, the demand of consumers for products rich in protein is of significant growth. Due to its structure in tissues, protein is considered an essential nutrient for maintenance and growth. It is well known that dairy foods differ from plant-based milk alternatives in their composition. In addition to protein content, nutrients in milk and plant-based beverages vary greatly in composition and content, such as: Calcium, fiber and fat. The nutritional quality of dairy protein sources depends on both their amino acid composition and bioavailability. Indeed, dairy products are considered to be excellent sources of proteins with high Digestible Indispensable Amino Acid Score (DIAAS) values varying from 100 to 120. However, plant proteins are considered to have generally lower essential amino acid contents and lower DIAAS values than dairy proteins. For example, pea and rice proteins are known to have medium and lower DIAAS with values of 62 and 47, respectively. The present review is dedicated to study the nutritional quality of animal and plant-based milk alternatives, where a focus on protein composition and amount are determined.

## 1 Introduction

In recent years, consumers have increasingly opted for plant-based diets (1, 2). In fact, according to Battisti et al. (3), plant-based milk alternatives are one of the emerging areas in the food industry, as the consumption of plant-based milk alternatives has increased significantly and is rapidly gaining popularity, mainly due to its nutritional value and numerous positive effects and health benefits to humans (4). Given that approximately 65% of the world’s population has reduced lactose digestibility and allergies, reliance on plant-based milk alternatives has emerged as an ideal alternative to meet the daily nutritional needs of these consumers.

In this context, both research and industry are interested in developing plant-based milk alternatives as an alternative to animal milk products. The market is expected to reach $66.9 billion by 2030, according to a new report from Grand View Research, Inc. as indicated in previous research studies (5, 6). As depicted by Pritulska et al. (6), plant-based milk alternatives could be produced from nuts (almond milk), grains (oat milk), legumes (soy milk), seeds (hemp milk) and so on.

Numerous research studies were conducted to determine the quality, functionality, and nutritional properties of plant-based milk alternatives. For example, Le Roux et al. (7) depicted that plant-based milk alternatives produced with pea and faba beans presented protein digestibility varying between 51–66 and 42–73% for static and dynamic digestion systems, respectively. In another approach, Khalesi and FitzGerald (8) blended soybean or pea protein at a level of 25% with animal milk protein and observed an amelioration in the digestibility level since soy and animal milk protein blend was found to be digested in the gastric phase, while pea and animal milk protein blend was mainly digested during the intestinal phase.

Despite the popularity of plant-based milk alternatives, these products sometimes presented some unpleasant sensory notes such as beany off-flavor, chalky or grainy mouthfeel, darker appearance, instability manifested by liquid separation (9, 10). To counteract these disadvantages and increase the acceptability of plant-based milk alternatives, flavorings and stabilizers are used in the formulation of plant-based milk alternatives (11). However, the use of additives in the formulation of plant-based milk alternatives may lead to concerns among consumers who are increasingly scrutinizing the nature of ingredient lists (12). To address this problem, recent research studies have been conducted by combining different plant proteins such as soy and almond milk blend, oat, and cashew blend, and flaxmilk and pea protein allowing to produce products with a high nutritional value (12). Therefore, this paper aims to provide a comprehensive review of the quality of plant-based milk alternatives compared to animal dairy products by focusing on protein.

## 2 Milk and milk analogs in terms of their composition in protein and digestibility

Dairy milk is considered to be a source of protein, fat, mineral especially calcium and phosphorus, and several other micronutrients (Table 1). For the production of plant-based milk alternatives, different ingredients such as: (i) vitamins A and D, minerals and so on (16); and (ii) sugars, flavors, are used to improve taste and texture, thus affecting the overall health profile.

**Table 1:** Composition of bovine milk compared to some plant-based milk alternatives.

	Macromolecules			Minerals		References
Milk type	Protein (%)	Fat (%)	Carbohydrates (%)	Ca (mg/100 g)	P (mg/100 g)	
Bovine milk	2.9–6	3.4–6.4	3.20–5.40	122–134	119–121	(13)
Almond milk	1.9–2.50	3.20–3.60	4.30–4.70	13.05–13.15	75.03–75.33	(14)
Soy milk	3.82–3.98	3.1–4.3	4.64–4.92	4–5.4	49–62.6	(14)
Rice milk	0.28–1.26	0.97–1.11	9.41–12.7	118–121.35	55.91–56.86	(15)
Coconut milk	0.59–2	4.12–6	3.75–9.41	176–178.1	240–256.35	(15)

Protein is fundamental to maintain human body function. The nutritional quality of proteins is affected by their amino acid composition and bioavailability. The protein level is calculated using the nitrogen conversion factor, depending on the protein’s origin. Table 2 expressed the nitrogen to protein conversion factor for animal and plant protein, while Table 3 indicated the amino acid composition varied according to the plant-based milk alternatives (25–27).

**Table 2:** Nitrogen to protein conversion factor for animal and plant protein.

Protein type	Factor	Reference
Milk	6.38	(17)
Almond	5.18	(18)
Rice	5.95	(17)
Soybean	5.71	(17)
Coconut	5.31	(19)

**Table 3:** Comparative overview of some amino acid profile of bovine milk with commercially nondairy plant-based milk alternatives.

	Some amino acids (mg/100 g)							References
Milk type	Lysine	Methionine	phenylalanine	tryptophan	Leucine	Histidine	Valine	
Bovine milk	49–96	17–27	38–56	n.d.	90–108	15–26	33–53	(5, 20)
Soy bean milk	0.88–3.92	0.31–0.85	1.86–2.79	0.3–0.8	2.94–4.24	0.55–1.49	1.32–2.59	(5, 21)
Almond milk	36.2–57.4	27.1–27.95	50.9–50.55	13.9–13.98	83.2–83	21.8–25.7	38.3–73.6	(5)
Rice milk	118.4–179.4	155.6–168.9	393.3–448.5	n.d.	496.9–585.2	186.6–206.6	306.2–375.2	(5, 22)
Peanut milk	36.75–36.7	n.d.	n.d.	30.02–30.3	64.5–64.3	27.2–27.73	32.63–32.79	(5, 23)
Coconut milk	3.50–5.1	1.2–2.9	2.7–5.9	3.20–3.30	3.9–6.5	1.8–1.9	3.5–7.5	(5, 24)

n.d., not determined.

The digestible indispensable amino acid score (DIAAS) of some plant-based milk alternatives has recently determined by Khamzaeva et al. (27) (Table 4). For cow’s milk, all DIAAS were higher than 100% with the lowest one (117%) for tryptophan and the highest one (198%) for histidine. Lower DIAAS values were determined for individual amino acids for the other plant-based beverages. Indeed, for soy plant-based beverages, the lowest DIAAS values (111%) was noted for valine and tryptophan and the highest one (164%) for histidine. Again, all DIAAS values for soy beverages were higher than 100%. Regarding oat beverages, histidine and lysine presented, respectively, the highest (183%) and the lowest (73%) DIAAS values, respectively. For the oat almond beverages, lysine, threonine and tryptophan presented the lowest DIAAS values, respectively: 34, 93 and 94%. The highest DIAAS values was noted for Histidine with 187%.

**Table 4:** Digestible indispensable amino acid score (DIAAS)^a^ ratio for Histidine, Threonine, Valine, Isoleucine, Leucine, Lysine and Tryptophan in soy, oat, and almond plant-based beverages and cow’s milk (27).

	DIAA reference ratio						
	Histidine	Threonine	Valine	Isoleucine	Leucine	Lysine	Tryptophan
Cow’s milk	1.98	1.48	1.35	1.51	1.38	1.60	1.17
Soy beverage	1.64	1.39	1.11	1.47	1.14	1.24	1.11
Oat beverage	1.83	1.17	1.24	1.30	1.18	0.73	0.95
Almond beverage	1.87	0.93	1.08	1.26	1.10	0.34	0.94

^a^The DIAA reference ratio was calculated by dividing the content of the indispensable amino acid by the reference pattern of the respective amino acid (27).

### 2.1 Soybean milk

The production and consumption of soybean milk have increased significantly over the last two decades due to its nutritional value and health benefit (25, 26). For example, besides the absence of lactose and cholesterol in soy milk, its protein composition is quite similar to that of cow milk (27).

According to the United States Department of Agriculture Food Composition Databases, soybean milk contains a protein level of 3.65 g/100 g. In order to increase the bioavailability of bioactive compounds present in soybean milk, a fermentation process could be applied. Indeed, it has been reported that the fermentation process reduced anti-nutritional factors (proteinase- inhibitors, phytic acid, urease, oxalic acids) and increase the bioavailability of bioactive components (28). The authors explained this trend by the fact that during the fermentation process, micro-organisms break down complex organic substances into simpler molecules increasing the number of free isoflavones and peptides (28). In another approach, Sanjukta and Rai (29) fermented soybean with *B. subtilis MTCC5480* and observed a higher amount of free amino acids level due to the protein hydrolysis. The authors mentioned that *B. subtilis* increase the free radical scavenging property to an appreciable level and inhibits angiotensin I-converting enzyme resulting in decreasing blood pressure level. Recently, Battisti et al. (3) analyzed 15 different commercial soy milk using a label-free quantitative proteomics approach and found different levels of essential amino acids and non-essential amino acids. The authors depicted a relative lower amount of histidine, methionine, tryptophan and cysteine in soy milk and recommended the necessity of fortifying commercial soy milk with these amino acids. The obtained results are confirmed, recently, by others who depicted the absence of tryptophan in soybean grain (27).

### 2.2 Almond milk

Almond is considered one of the “brain-foods” since it is considered to promote mental alertness, concentration, recall skills, memory and helps to get good sleep when taken at night (30). Patients who are suffering from lactose intolerance are advised to consume almond milk instead of soy milk (31). Recently, Ashkanani (32) compared the nutritional quality of almond and oat milk and found that the former was more effective to increase protein level among others. Ashkanani (32) depicted that the *in vitro* digestion of almond proteins by pepsin led to the destabilization and coalescence of almond oil bodies that did not significantly affect the rate of protein delivery to the small intestine.

In a different approach, Wang et al. (33) determined the DIAAS of almond milk compared to cow milk. It is well known that the higher the DIAAS score, the greater the quality of the protein material in the food. The authors depicted a DIAAS of 0.39 and 1.45 for almond and cow milk, respectively indicating the higher digestibility of the former milk. The same authors used another universal score called PDCAAS and again found that cow milk scored higher than almond milk (1 vs. 0.4, respectively). One of the main conclusions of their study was that almond milk is not a substitute for cow’s milk because of its lower DIAAS value.

Almond is ranked as fourth among other tree nuts allergy that could be presented as mild such as simple oral allergy and complex as fatal anaphylaxis. Among allergy compounds, amandin is the major protein in almond, legumin, and pruning. The amandin allergen is highly resistant to heat treatments but sensitive to pepsin enzyme (34). As for soy bean milk, the application of mechanical and fermentation treatments removed easily allergen proteins allowing almond milk to make its position among other plant-based milk alternatives substitutes in the market.

### 2.3 Rice milk

Rice milk is made primarily from ground rice and water. It is easy to digest, and suitable for allergy sufferers. Like other plant-based beverages, rice milk presents a creamy texture that resembles dairy milk (35). Although rice contains a relatively high level of proteins (10%), it suffers from the absence of threonine and lysine. On the contrary, it contains significant amounts of ferulic acid, sinapic acid and p-coumaric acid. The most abundant amino acids in black rice milk are leucine, glutamic acid, serine, and aspartic acid (36). The low protein content in black rice milk contributed to the low number of amino acids in agreement with the findings of others (35).

It has been reported that soaking is effective in increasing the minerals and vitamins (B6 and B12), insoluble fiber and bioactive components in rice (37). As for soy and almond milk, fermentation with the use of lactic acid bacteria breaks down the anti-nutritional factors and enhances calcium, magnesium, and iron levels, and helps in the digestion and immunity of other internal organs (37).

### 2.4 Coconut milk

Coconut milk is prepared by a mechanical method that starts by shelling the nut and separating the meat, which is cleaned and grated. Mixing with warm water to extract oil, milk, and aromatic components (38). Different parameters such as grinding time and incubation time present a major impact on coconut milk yield production. Coconut milk contains protein, fat, carbohydrates, minerals (calcium, phosphorus, and potassium), and vitamins (vitamins B1, B3, B5, and B6, C, E) (39). Coconut protein presents a large number of essential amino acids, which are more easily digested and absorbed with a DIAAS value of 0.79 versus 1.45 for cow milk (40).

Thaiphanit and Anprung (41) produced yoghurt samples with different ratios of cow and coconut milk (100:0, 80:20, 60:40, 50:50, 40:60, 20:80, 0:100). The authors found that producing yoghurt with cow and coconut blends is more nutritious than the ordinary one and suggested more exploration of the use of coconut and cow blend milk for the production of yoghurt.

### 2.5 Oat milk

Oat presents nutritional components including phenolic compounds, saponin, sterol, phytic acid and other anti-oxidant components. Oat contains various fiber components such as polysaccharides, oligosaccharides, lignin. Plant-based beverages containing lentils and peas or just adding peas to oat drinks increase the concentration of amino acids (42). The authors found that the most ideal mixture to obtain a complete amino acid composition was obtained with: (i) a raw material containing 1.1% oat protein, 1.5% each pea and lentil protein; (ii) 1.1% oat protein, 2.9% pea protein; (iii) 0.8% oat protein, 1.1% pea protein, and 2.1% lentil protein. These mixtures were found to significantly increase the amounts of phenylalanine, leucine, and threonine, and to a lesser extent isoleucine, valine, methionine, histamine and lysine. One of the main conclusions of their study is that most plant-based beverages made from single-plant ingredients do not have an amino acid profile that meets human needs.

As observed for other plant-based milk alternatives, the fermentation process induces the formation of active ingredients improving thus the quality of plant-based milk alternatives, plant based dairy products (43). In this context, germinated oat beverages fermented with *Lactobacillus reuteri*, *Lactobacillus plantarum* B28, and *Streptococcus thermophilus* was found to present health benefits for consumers (44, 45).

## 3 Conclusion

Plant-based milk alternatives will continue to be an important research area in the new product development category of food science and technology by setting a more strategic direction for innovation and next-generation protein blends. Plant-based milk alternatives meet the changing consumer behavior toward novel plant-based milk alternatives, the scientific community expects continuous efforts to improve plant-based milk alternatives quality through R&D activities and technological interventions. It is noted that deep and continuous research studies should be realized in the next years to ameliorate the nutritional quality of plant -based milk, particularly in their composition in amino acids. This could be achieved by combining different plant proteins that induce an amelioration in the composition of amino acids of plant-based milk alternatives. In addition, research on plant-based milk alternatives should be deepened regarding the amelioration of their organoleptic properties and the prolongation of their shelf life. This can be achieved by inactivating plant enzymes using new process techniques such as high-pressure treatment, pulsed electric fields, ohmic heating and cold plasma.

## Author contributions

RK: Conceptualization, Investigation, Methodology, Project administration, Supervision, Validation, Writing – review & editing. IB: Conceptualization, Writing – original draft.
